# Computational Identification and Modeling of Crosstalk between Phosphorylation, *O*-β-glycosylation and Methylation of FoxO3 and Implications for Cancer Therapeutics

**DOI:** 10.3390/ijms13032918

**Published:** 2012-03-05

**Authors:** Azeem Mehmood Butt, Dandan Feng, Muhammad Idrees, Yigang Tong, Jun Lu

**Affiliations:** 1 Division of Molecular Virology, National Centre of Excellence in Molecular Biology (CEMB), University of the Punjab, Lahore 53700, Pakistan; E-Mails: azeem@cemb.edu.pk (A.M.B.); idreeskhan@cemb.edu.pk (M.I.); 2 Cancer Biotherapy Ward, Beijing YouAn Hospital, Capital Medical University, FengTai District, Beijing 100069, China; E-Mail: fengdandan83@gmail.com; 3 State Key Laboratory of Pathogen and Biosecurity, Beijing Institute of Microbiology and Epidemiology, Beijing 100071, China; E-Mail: tong62035@gmail.com

**Keywords:** FoxO3, FoxO, *in silico*, posttranslational modifications, phosphorylation, *O*-β-glycosylation, methylation, cancer, Yin Yang sites

## Abstract

FoxO3 is a member of the forkhead class of transcription factors and plays a major role in the regulation of diverse cellular processes, including cell cycle arrest, DNA repair, and protection from stress stimuli by detoxification of reactive oxygen species. In addition, FoxO3 is a tumor suppressor and has been considered as a novel target for cancer therapeutics. Phosphorylation of FoxO3 via the AKT, IKK, and ERK pathways leads to deregulation, cytoplasmic retention, degradation of FoxO3 and favors tumor progression. Identification of the amino acid residues that are the target of different posttranslational modifications (PTMs) provides a foundation for understanding the molecular mechanisms of FoxO3 modifications and associated outcomes. In addition to phosphorylation, serine and threonine residues of several proteins are regulated by a unique type of PTM known as *O*-β-glycosylation, which serves as a functional switch. We sought to investigate the crosstalk of different PTMs on the FoxO3 which leads to the onset/progression of various cancers and that could also potentially be targeted as a therapeutic point of intervention. A computational workflow and set of selection parameters have been defined for the identification of target sites and crosstalk between different PTMs. We identified phosphorylation, *O*-β-GlcNAc modification, and Yin Yang sites on Ser/Thr residues, and propose a potential novel mechanism of crosstalk between these PTMs. Furthermore, methylation potential of human FoxO3 at arginine and lysine residues and crosstalk between methylation and phosphorylation have also been described. Our findings may facilitate the study of therapeutic strategies targeting posttranslational events.

## 1. Introduction

Since the initial discovery of the *forkhead* gene in *Drosophila melanogaster*, more than 100 forkhead genes and 19 human subgroups that extend from FOXA to FOXS are now known to exist. FoxO transcription factors belong to the “O” (“other”) class of the FOX superfamily. In mammals, four members of FoxO have been identified: FoxO1, FoxO3, FoxO4, and FoxO6 [1]. These transcription factors are found to be distributed throughout the body. However, expression of FoxO proteins is not the same across tissues, suggesting that individual FoxO proteins have specific cellular functions, thereby, forming the most divergent FOX subfamily due to a unique five amino acid (GDSNS) insertion immediately prior to helix H3 within the forkhead domain. This motif is directly involved in sequence specific interaction with DNA binding sites [2].

Human FoxO3 (or FoxO3a) plays a critical role in modulating diverse cellular processes, such as metabolism, differentiation, and transformation in animal cells. FoxO3 can act as both transcriptional activators and repressors through binding interactions between target DNA and the DNA binding domain (DBD). The FoxO3 gene was first identified at the t(6;11) (q21;q23) chromosomal translocation from an acute myeloid leukemia patient [3]. Since then, several studies have shown direct correlation between FoxO3 expression and tumor progression. Low expression and cytoplasmic retention of FoxO3 was found to be associated with the development of ovarian and breast cancer, and leukemia and correlated strongly with poor patient survival. In contrast, FoxO3 overexpression inhibited tumor growth *in vitro* and tumor size *in vivo* in several cancers such as breast, lymphomas, and hemangiomas [4–6]. FoxO3 promotes cell cycle arrest in mouse myoblastic cell lines through modulation of growth arrest and DNA damage response proteins [7]. Activation of FoxO3 antagonizes cell proliferation and promotes apoptotic cell death in chronic myelogenous leukemia cell lines. Therefore, during tumor development, inhibition of FoxO3’s transcriptional activity promotes cell transformation, tumor progression, and angiogenesis. The loss of FoxO3 activity in association with c-myc, p27, and NF-KB can result in cell cycle induction and malignant transformation of mouse cells in the presence of oncogene activation [8,9]. Pro-apoptotic effects and the ability to block cell cycle progression suggest FoxO3 is an ideal therapeutic target to control tumorigenesis. Collectively, all these studies indicate that FoxO3 is a bonafide tumor suppressor and that deregulation of FoxO3 activity is a major factor in cancer progression. In general, FoxO3 activity is regulated by different posttranslational modifications (PTMs) such as phosphorylation, acetylation, and ubiquitination [10,11]. These different PTMs allow FoxO3 to function in various cellular activities by changing its subcellular location, molecular half-life, or DNA-binding activity [12–14].

It is well known that different PTMs modulate the function and activity of target proteins by inducing structural changes and changes in cellular localization. PTMs form a complex regulatory network with characteristics of a sophisticated language, and such a network is fundamental to normal development as well as disease pathogenesis [15,16]. It is also recognized that often one PTM may enhance or prevent another PTM, resulting in their interplay regulating diverse molecular processes. One such example is the interplay between phosphorylation and *O*-β-GlcNAc modifications on the same or neighboring Ser/Thr residues, also known as “Yin Yang” sites [17]. *O*-GlcNAcylation is a ubiquitous and highly dynamic modification that is regulated by *O*-GlcNAc transferase (OGT: adds *O*-GlcNAc to protein backbone) and *O*-GlcNAcase (OGN: removes *O*-GlcNAc from protein backbone) on serine and threonine residues of nuclear and cytoplasmic proteins. *O*-GlcNAcylation plays a critical role in protein folding, localization and trafficking, solubility, antigenicity, biological activity, and half-life, as well as cell-cell interactions. Since discovery of interplay between phosphorylation and *O*-β-glycosylation, several proteins have been computationally and experimentally studied for this interplay and it has been proposed as a point of intervention for functional modulation [18–20].

In addition to the interplay between phosphorylation and *O*-β-glycosylation, other PTMs also show interactions, such as the interplay between methylation and phosphorylation [21]. Protein methylation is an important and reversible type of PTM that governs cellular dynamics and plasticity. Protein methylation can modify the nitrogen atoms of either the backbone or side chain (*N*-methylation) in several types of amino acids such as Lys, Arg, His, Ala, and Asp. However, most protein methylation studies have focused on Lys and Arg residues. Depending upon cellular environment, Arg and Lys can be methylated once, twice or thrice by Peptidyl Arginine Methyltransferases (PRMTs) and Histone Lysine Methyltransferases (HKMTs), respectively [22]. Many studies have started to reveal the important role of Arg methylation in different cellular processes such as RNA processing, transcriptional regulation, signal transduction, DNA repair, and protein-protein interactions [23–25]. In addition, reports have started to identify and characterize the regulatory crosstalk between arginine methylation and phosphorylation for proteins such as RIP140 [26] and STAT6 [27]. However, understanding the functional changes induced is still remains to be elucidated.

Unbiased identification of PTM sites by experimental methods is expensive and time consuming. In contrast, advance computational algorithms trained on available experimental data can provide functional candidates and help narrow down the experimental efforts are much more desirable for their effectiveness at identifying potential PTM target sites in proteins of interest. The aim of this study was to identify PTMs sites and their crosstalk with respect to regulating human FoxO3. An *in silico* workflow comprised of several machine learning algorithms was adopted for this purpose. Next, based on available experimental data and findings of this computational analysis, crosstalk between (a) phosphorylation and *O*-β-glycosylation at Yin Yang sites; and (b) between phosphorylation and methylation at neighboring Ser/Thr/Tyr and Arg residues have been described.

## 2. Materials and Methods

### 2.1. Analysis Data

The amino acid sequence of human FoxO3 was retrieved from the SWISS-PROT database [28] using the primary accession number O43524 and the entry name, FOXO3_HUMAN. A BLASTP [29] amino acid sequence homology search was performed using the National Center for Biotechnology Information (NCBI) database using default parameters for all known sequences. The sequences selected from various organisms were based on higher bits score, and *E*-values ≤ 0. The selected sequences, including the query, were aligned using ClustalW2 [30] to determine the level of conservation across various species and to locate conserved Ser/Thr/Tyr/Arg and Lys residues. The five selected target sequences for the FoxO3 protein originated from *Sus scrofa* (RefSeq. NP_001129431.1), *Rattus norvegicus* (RefSeq. NP_001099865.1), *Mus musculus* (RefSeq. NP_062714.1), and *Bos taurus* (RefSeq. XP_615634.2).

### 2.2. Prediction of Phosphorylation, Kinases Activity, and Solvent Accessibility of Human FoxO3

The phosphorylation potential of human FoxO3 was predicted by NetPhos 2.0 [31]. This program is based on a artificial neural network approach and predicts the potential phosphorylation sites on each Ser, Thr, and Tyr residue, by calculating a score for phosphorylation potential. NetPhos 2.0 uses a threshold value of 0.5 for any Ser, Thr, and Tyr to indicate a potential phosphorylation site.

ScanSite 2.0 [32] was used to predict the activity of various kinases on phosphorylation sites. This program has the ability to not only predict kinase activity, but also determine surface accessibility (SA) for each amino acid residue. The amino acid sequence at each candidate site (seven amino acid residues on both sides of a potentially modified amino acid) was evaluated according to the preference of specific protein kinases. The program then indicates the percentile ranking of the candidate motif with respect to all potential vertebrate motifs in the SWISS-PROT database. Additionally, the SA value for each amino acid was determined. If SA ≥ 0.5, the amino acid was predicted to be exposed on the protein surface and accessible for PTM.

### 2.3. Prediction of *O*-β-GlcNAc Modifications and Yin Yang Sites in Human FoxO3

*O*-β-GlcNAc modifications of human FoxO3 were predicted using YinOYang 1.2 [33]. YinOYang calculates the *O*-β-glycosylation potential for all Ser and Thr residues in a protein sequence, and crosschecks these sites against NetPhos 2.0 predictions for potential phosphorylation sites to determine Yin Yang sites with high potential for both modifications.

### 2.4. Prediction of Methylation in Human FoxO3

For the prediction of methylation potential at Arg and Lys residues, we used a combination of three prediction algorithms; MeMo v2.0 [34], BPB-PPMS [35] and MASA [36]. BPB-PPMS is based on Bi-profile Bayes combined with support vector machines (SVMs), whereas MASA predictions are calculated on the basis of accessible surface area surrounding the modification sites.

### 2.5. Selection Parameters for Posttranslational Modifications Residues

Despite the fact that algorithms used in this study are well-trained on experimental data and the training datasets are continuously updated, identification of false-positives can happen. This can be avoided by keeping in consideration multiple selection parameters based on physiochemical, molecular, structural, and evolutionary characteristics of proteins. Therefore, for the purpose of avoiding false-positive predictions, we defined set of rules which are as follows; (i) comparison of predicted sites with previously known experimentally verified sites of PTMs. The information on experimentally verified PTM sites was taken from the Phospho.ELM [37] and Swiss-Prot databases. The Phospho.ELM resource is a relational database designed to store *in vivo* and *in vitro* phosphorylation data extracted from the scientific literature; (ii) Evolutionary conservation status of the predicted sites was inferred from the ClustalW2 multiple sequence alignment results, as it is known that residues which are evolutionary conserved tend to be more likely involved in functional activities; (iii) Whether the predicted site is structurally accessible to be targeted based on SA values obtained via ScanSite prediction algorithm. The same set of rules were applied for the evaluation of predicted phosphorylation, *O*-β-glycosylation, Yin Yang, and methylation sites.

## 3. Results

### 3.1. Human FoxO3 Possess Multiple Phosphorylation Sites

**Figure 1:**
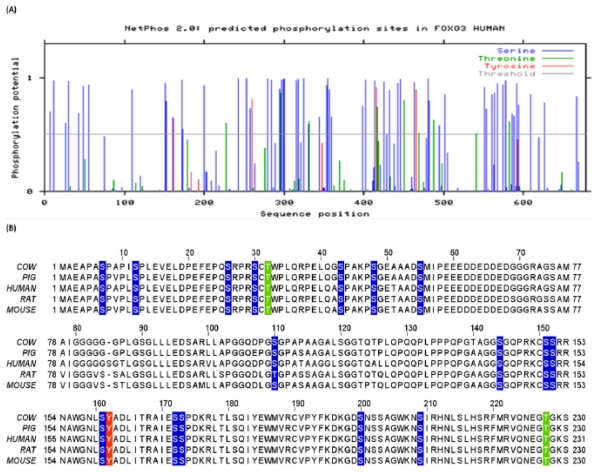

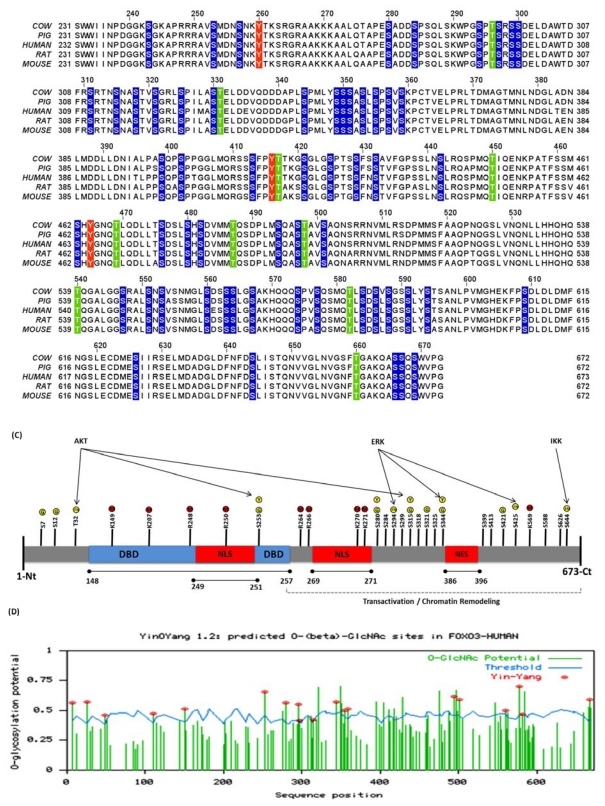
(**A**) Graphical representation of potential sites (Ser, Thr, and Tyr) for phosphorylation in human FoxO3 as inferred from NetPhos 2.0. The blue vertical lines show the potential phosphorylated Ser residues; the green lines show the potential phosphorylated Thr residues; the red lines show the potential phosphorylated Tyr residues. The light gray horizontal line indicates the threshold for modification potential; (**B**) Multiple sequence alignment of human FoxO3 and closely related species. Conserved Ser, Thr, and Tyr residues are highlighted in blue, green, and red colors respectively; (**C**) Schematic representation of primary structure of human FoxO3. Experimentally verified phosphorylation sites are shown along with computationally predicted *O*-β-glycosylation and Yin Yang sites. *O*-β-glycosylation sites (G in yellow circle), positive Yin Yang sites (Y in yellow circle), and false-negative Yin Yang sites (FN in yellow circle). In addition methylation sites (M in red circle) are also shown. DBD: DNA binding domain (amino acids: 148–257); NLS: Nuclear localization signal (amino acids: 249–251; 269–271); NES: Nuclear export sequence (amino acids: 386–396); transactivation/chromatin remodeling domain (amino acids; 258–673); (**D**) Graphical representation of potential sites for *O*-β-GlcNAc modification on Ser and Thr residues in human FoxO3 as inferred from YinOYang 1.2. The green vertical lines show the *O*-β-GlcNAc modification potential of Ser/Thr residues and the light blue horizontal wavy line indicates the threshold for modification potential. The positively predicted Yin Yang sites are shown with red asterisk at the top. The light blue horizontal wavy line indicates the threshold for modification potential.

The prediction of generic phosphorylation sites in human FoxO3 was performed using neural network algorithm that is implemented via NetPhos 2.0 server. A total of 72 sites were identified with high potential for phosphorylation. Amongst these 72 phosphorylation sites, 59 were Ser, 9 were Thr, and 4 were Tyr (Figure 1A and Table 1). Next, we evaluated each prediction based on selection parameters defined in the materials and methods section. As per selection criteria (i), we retrieved information about previously identified and experimentally validated phosphorylation residues of human FoxO3 from SwissProt and Phospho.ELM databases. This search led to retrieval of 21 amino acid residues (20 Ser, 1 Thr) (Table 1). In the next step, these residues were tallied with the predicted sites. It was observed that in addition to identification of several novel phosphorylation sites, our predictions results also included phosphorylation sites from previous experiments, thus supporting the strategy of a computational approach (Table 1). As per selection criteria (ii); the level of conservation was inferred for each of the identified residues (Figure 1B). It was observed that all of the newly identified sites as well as experimentally verified sites from previous studies were evolutionary conserved among different species. From the schematic mapping of predicted phosphorylation sites using the primary structure of FoxO3, we observed several Ser/Thr/Tyr residues that were identified in this study and in previous experimental studies present in functionally important domains (Figure 1C).

### 3.2. Human FoxO3 Is a Target of Multiple Oncogenic Kinases

Although FoxO3 is phosphorylated under different conditions and several kinases have been identified that induce phosphorylation under various cellular environments, we suspect there are several additional kinases which act on FoxO3 but that have not yet been discovered. Similarly, there are experimentally verified phosphorylation sites of human FoxO3 for which kinases have not yet been identified. This includes Ser280, Ser284, Ser299 and Ser421 [37]. Identification of kinases responsible for phosphorylation holds the key to generate a complete activity map of FoxO3 as well as for the development of kinase inhibitors. Therefore, once generic phosphorylation residues were identified, we sought to predict which kinases have the potential to phosphorylate these residues. ScanSite was used to predict the activity levels of kinases for FoxO3 and the results compared with NetPhos predictions. Furthermore, as per selection criteria (iii), SA values obtained for each phosphorylation site by ScanSite and were used to determine the degree of environmental exposure of each residue. Based on ScanSite predictions and SA values, we were able to identify most of the experimentally verified kinase-dependent phosphorylation sites from previous studies (Table 1), and to predict the activity of several additional kinases on the same and newly identified Ser, Thr, and Tyr residues. For instance, Ser280 was identified as a substrate for CK2 and GSK3, Ser284 as a substrate for ERK, and Ser421 as a substrate for CK1 (Table 1). Since FoxO3 is known to be phosphorylated by AKT and ERK, we investigated if there were any additional AKT and ERK target sites in human FoxO3 not yet identified. Ser12 and Ser413 were identified as target sites of AKT whereas; Ser12, Ser284, Ser325, Ser355, and Ser402 were identified as target sites of ERK (Table 1). Again, each of the residues identified were found to be conserved among closely related species and accessible to kinases for phosphorylation as per SA values.

### 3.3. Cdk5 Associated Phosphorylation of Human FoxO3

Five sites (Ser43, Ser173, Ser294, Ser355, and Ser425) identified as target sites of Cdk5 during this study are of particular interest. Among these sites, Ser294 and Ser425 are already known to be phosphorylated by an oncogenic kinase, ERK that leads to the nuclear exclusion and degradation of FoxO3 in several cancers [5] (Table 1). Cancer metastasis correlates directly with Cdk5 expression. In two different studies on prostate and pancreatic cancer metastasis, inhibition of Cdk5 expression inhibited prostate [47] and pancreatic cancer metastases in experimental models [48]. Presently, no experimental data is available regarding the ability of Cdk5 to phosphorylate FoxO3. However, progression of prostate and pancreatic cancers due to deregulation and nuclear translocation of phosphorylated FoxO3 have been documented [49,50]. Therefore, based on our findings, we suggest that Cdk5 may also play a role in deregulation of FoxO3 by inducing phosphorylation at Ser43, Ser173, Ser294, Ser355, and Ser425 and warrants experimental studies. Each of these sites was found to be conserved and showed high potential for phosphorylation. It is not yet known if Cdk5 has a preference for phosphorylating single or multiple sites. However, considering SA values, Ser173 had highest SA value, *i.e.*, 3.5 as compared to other sites (Table 1).

### 3.4. Phosphorylation of Human FoxO3 at Tyrosine Residues

In addition to phosphorylation at Ser/Thr residues, phosphorylation of Tyr residues is another important event that plays a critical role in signal transduction and is associated with cell proliferation, survival, apoptosis, mobility, and adhesion. Tyrosine-phosphorylated proteins (also known as phosphotyrosine proteins, pTyr) include a wide range of signaling molecules, such as receptor tyrosine kinases, adapter proteins, and scaffold proteins, which are known to be involved in the cancer metastatic process [51]. Aberrant expression and activity of pTyr proteins in cell-signaling have been reported in various human cancers [52,53]. The phosphorylation potential of FoxO3 at its Tyr residues is not yet known. By using neural network based algorithms and selection parameters, we propose that human FoxO3 has strong potential for phosphorylation at its Tyr residues including Tyr162, Tyr260, Tyr416, and Tyr465 (Table 1).

The data obtained from this analysis of kinases activity and phosphorylation shows that human FoxO3 has many “hot spots” for several different kinases and serves as a valuable source for further experimental studies. Although, ScanSite was not able to predict SA values and kinases for some sites, these sites were still considered to be positive predictions based on their phosphorylation potential, conservation status, and structural categorizations.

### 3.5. *O*-β-GlcNAc Modifications and Yin Yang Sites in Human FoxO3

YinOYang 1.2 was used for the prediction of sites of *O*-β-GlcNAc modifications and potential Yin Yang sites. A total of 41 Ser/Thr residues were identified with very high potential for *O*-β-GlcNAc modification. Amongst these, 33 were Ser and 8 were Thr. The results from YinOYang were crosschecked against NetPhos predictions and out of those 41 *O*-β-glycosylation sites, 17 Ser and 2 Thr residues were identified as positive Yin Yang sites (Figure 1D and Table 1). These individual *O*-β-glycosylation and positive Yin Yang sites were found to be evolutionary conserved and distributed across the total length of FoxO3 at several functionally important regions, such as the DBD, and phosphorylation motifs of AKT, NLS, and NES as inferred from multiple sequence alignment and primary structure analysis respectively (Figure 1B,C). Among the 19 positive Yin Yang sites, several residues had previously been experimentally verified as substrates for different kinases for phosphorylation (Table 1). For instance, Ser253 and Ser315, which are part of the AKT phosphorylation motif, and Ser344 that is phosphorylated by ERK, were predicted as positive Yin Yang sites. These results strongly suggest that human FoxO3 proteins have high potential for *O*-β-GlcNAc modifications on multiple Ser/Thr residues.

### 3.6. Identification of False-Negative Yin Yang Sites in Human FoxO3

In addition to the positive Yin Yang sites, some of the Ser and Thr residues suggest a very high potential for either *O*-β-GlcNAc modification or phosphorylation, or show a potential very close to the specific threshold value as predicted by existing methods. Such sites are termed as false-negative Yin Yang sites, when they are evolutionary conserved, as on these sites OGT and kinases may have an equal accessibility for inducing PTMs of interest [20]. Following this observation and previously mentioned selection parameters, we identified four false-negative Yin Yang sites in human FoxO3, residues Thr32, Ser294, Ser425, and Ser644 (Table 1).

### 3.7. Methylation Potential of Human FoxO3

Two approaches “similarity search” and “machine learning prediction algorithms” were used for identifying methylation sites. Initially, multiple sequence alignment between mouse FoxO1 and human FoxO3 protein was performed (data not shown), indicating that the Arg248 and Arg250 from mouse FoxO1 corresponded to Arg248 and Arg250 of human FoxO3. Next, based on consensus predictions from different algorithms, nine novel methylation sites among which 4 were Arg and 5 were Lys were identified, including Arg248 and Arg250 (Table 2). Conservation status and schematic mapping was performed and each residue was found to be evolutionarily conserved and part of different functional regions of the protein (Figure 1C and Table 2). For instance, Arg248 and Arg250 were found to be part of the overlapping DBD and NLS regions located in close proximity to Ser253 (substrate of AKT). Arg264 and Arg266 resided within a transactivation domain (Figure 1C and Table 2).

Our results also included 5 Lys residues with high potential for methylation (Table 2). Among the Lys residues identified, Lys207 was found to be part of a DBD, and a neighboring residue, Ser209, which is a substrate of STK4/MST1. The possibilities of one amino acid influencing the PTM status of other amino acid cannot be ruled out. Presently, the methylation potential at Lys residues in FoxO proteins is completely unknown. If FoxOs have the potential to be methylated at Lys residues, this suggests the presence additional regulatory mechanisms and potential crosstalk between acetylation and methylation on same Lys residues that is waiting to be explored.

## 4. Discussion

### 4.1. Crosstalk Between Phosphorylation and *O*-β-Glycosylation in Human FoxO3 Can Inhibit AKT, ERK, and IKK Pathways

In addition to other types of PTMs that modulate FoxO3 activity [11], two members of the FoxO family, FoxO1 and FoxO4, were recently recognized as *O*-β-GlcNAc modified proteins. In one study, the authors evaluated the potential of OGT derived *O*-β-GlcNAc modifications on FoxO1 protein in diabetes. Interestingly, it was observed that *O-*β-GlcNAc on hepatic FoxO1 increased in diabetes. Furthermore, *O-*β-GlcNAc modifications regulated FoxO1 activation in response to glucose, resulting in a paradoxical increase in expression of gluconeogenic genes while concomitantly inducing expression of genes encoding enzymes that detoxify ROS. In the same study, the *O*-β-glycosylation potential of FoxO1 was evaluated by mutating AKT phosphorylation sites. This mutational strategy led to amplification of *O*-β-GlcNAc levels, indicating presence of a potential Yin Yang relationship [55]. Similarly, in another recent study, the authors demonstrated that *O*-β-GlcNAcylation enhanced FoxO4 transcriptional regulation in response to stress [56]. However, precise identification of Ser/Thr residues where *O*-β-GlcNAc modifications could take place has not yet been performed. Similarly, sites of crosstalk between phosphorylation and *O*-β-glycosylation, *i.e.*, Yin Yang sites remains to be determined in human FoxO3 and other members of FoxO family. Recently, we have reported potential crosstalk between phosphorylation and *O*-β-glycosylation of claudin-1, -3, and -4 proteins at Yin Yang sites [20]. Using a similar strategy, we investigated the *O*-β-glycosylation potential of human FoxO3 and identified 41 *O*-β-glycosylation sites in human FoxO3 (Figure 1D and Table 1). We next examined the phosphorylation and *O*-β-glycosylation crosstalk potential at each site and 17 Ser and 2 Thr residues were identified as positive Yin Yang sites. Based on selection parameters, four conserved and functionally important sites; Thr32 (substrate of AKT), Ser294 and Ser425 (substrates of ERK) and Ser644 (substrate of IKK) were identified as false-negative Yin Yang sites. These sites predicted to have high potential for crosstalk between phosphorylation and *O*-β-GlcNAc modifications based on the activity of kinases and OGT, respectively (Figure 1C and Table 1).

AKT, ERK, and IKK are the three oncogenic kinases most commonly activated in different types of human cancers. Studies have demonstrated a direct and dominating role of these kinases on human FoxO3 in comparison to other kinases currently known to phosphorylate FoxO3 [1,57,58]. Activation of the PIP3-AKT pathway via stimulation received by growth factors and insulin is a common event in several cancers, and leads to the activation and translocation of phosphorylated AKT into the cytoplasm. Once inside the nucleus, AKT actively phosphorylates three conserved residues (Thr32, Ser253 and Ser315) of human FoxO3 in a sequential manner. Phosphorylation of the forkhead domain residue, Thr32 by AKT, is a critical step and results in disruption of DNA binding, and the NLS and finally nuclear exclusion of FoxO3. Therefore, inhibiting the initial phosphorylation of Thr32 represents an attractive strategy to block FoxO3 phosphorylation. Unfortunately, the PIP3-AKT pathway is not the only reason for deregulation and nuclear exclusion of FoxO3, as observed in several cancers. Simultaneous and constitutive activation of multiple signal transduction pathways, such as the RAS-ERK and IKK/NF-KB pathways, is a common event that leads to cancer metastasis and resistance to clinical therapy [59]. The RAS-ERK pathway is known to play a pivotal role in differentiation, proliferation, and tumor progression. ERK directly interacts and phosphorylates three sites of human FoxO3 (Table 1). This in turn leads to FoxO3 degradation in the cytoplasm via the MDM-2-mediated ubiquitination proteasome pathway [5]. Similar to ERK, IKK also interacts physically with FoxO3 and phosphorylates Ser644, which leads to nuclear exclusion and proteasomal degradation. IKK is a central regulator of NF-κB that plays an important role in controlling cell proliferation, survival, the prevention of apoptosis and tumorigenesis [4]. Inhibition of ERK holds promising therapeutic potential as it was observed that a non-phosphorylated FoxO3-mimic mutant was more resistant to the interaction and degradation by MDM-2, resulting in strong inhibition of cell proliferation *in vitro* and tumorigenesis *in vivo* [5]. Cancer drugs such as Imatinib (for the treatment of chronic myeloid leukemia), cetuximab, lapatinib, gefitinib (for the treatment of breast, prostate, kidney and ovarian cancers) also target both AKT and ERK in order to inhibit phosphorylation of FoxO3 [60]. Therefore, it has been proposed in several studies, that inhibiting phosphorylation by AKT, ERK and IKK can potentially restore the function and prolong retention of FoxO3 in the nucleus. Unfortunately, resistance to chemotherapeutic agents is on the rise and poses a major problem in treating cancers [61]. In most cases, AKT, ERK and IKK appear to work independently and inhibition of one does not guarantee inhibition of FoxO3 phosphorylation by the other. Therefore, alternative strategies that can inhibit phosphorylation of FoxO3 on a large scale are desired.

Reciprocal regulatory events between phosphorylation and *O*-β-glycosylation are well documented for several proteins [19,20]. The recent identification of FoxO1 and FoxO4 as glycosylated proteins [55,56] and findings from our study (Table 1) further supports the fact that *O*-β-glycosylation may also regulate FoxO3 via interplay of kinases and OGT at Yin Yang sites. We propose the presence of a regulatory/therapeutic pathway between phosphorylation and *O*-β-glycosylation at the four false-negative Yin Yang sites. A growing body of evidences also suggests that dynamic cycling of *O*-β-GlcNAc is necessary for various cellular processes [17,19]. Therefore, increasing *O*-β-GlcNAc modification levels, either pharmacologically or via OGT overexpression, may induce inhibition of kinase-induced phosphorylation of targeted residues of FoxO3. This would provide a regulatory and therapeutic switch to control FoxO3 nuclear localization, gene regulatory activities, and cancer metastasis.

### 4.2. Crosstalk Between Phosphorylation and Methylation in Human FoxO3

**Figure 2:**
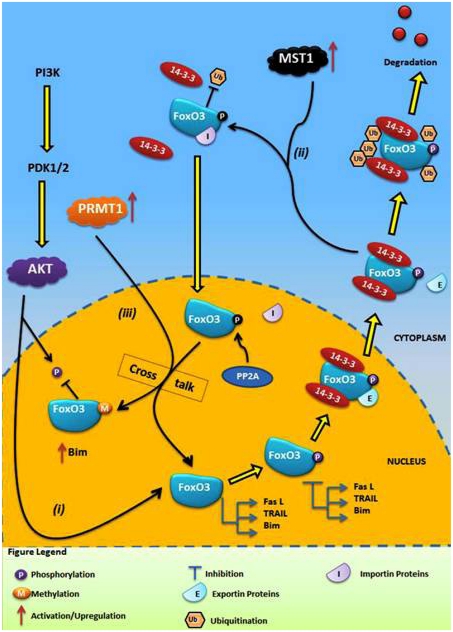
Graphical illustration of crosstalk between phosphorylation and methylation in human FoxO3. (**i**) Phosphorylation of FoxO3 via AKT at its target sites generates two binding motifs for 14-3-3 proteins. Binding of 14-3-3 proteins exposes NES of FoxO3. This complex is then rapidly transported out of the nucleus and retain within the cytoplasm, leading to degradation of FoxO3 by proteasomes; (**ii**) On the other hand, cellular oxidative stress also induces activation and upregulation of MST1 that actively phosphorylates and disrupts FoxO3-14-3-3 proteins complex leading to transport of FoxO3 back into the nucleus; (**iii**) Inside nucleus, PRMT1 methylates FoxO3 thereby masking AKT binding sites and preventing subsequent phosphorylation and nuclear exclusion.

An *in vivo* and *in vitro* study recently identified the methylation potential of mouse FoxO1, and demonstrated the presence of competitive interplay between methylation and phosphorylation. The authors identified two Arg residues at positions 248 and 250 in the AKT phosphorylation motif of mouse FoxO1. Methylation at these residues by PRMT1 competitively inhibited AKT-induced phosphorylation at Ser253 [54]. It is now well established that FoxO3 is a target of multiple PTMs and members of the FoxO family also exhibit interfamily homology at conserved sites. We therefore speculated that human FoxO3 can also be methylated, and exhibit potential methylation-phosphorylation interplay. Based on our current understanding of methylation of mouse FoxO1 and our computational analysis, we propose a model of FoxO3 regulation via crosstalk between methylation and phosphorylation. This functional crosstalk consists of three major events; (i) AKT induced phosphorylation of FoxO3 (ii) activation of mammalian Ste20-like kinase (MST1) in the cytoplasm in response to increased concentration of ROS and, (iii) an activation and increase in PRMT1 concentration in the nucleus. Collectively, these three events form the basis of competitive crosstalk between methylation and phosphorylation with FoxO3, as discussed below and shown graphically in Figure 2.

Oxidative stress induces activation of the PIP3-AKT pathway that ultimately leads to nuclear exclusion of FoxO3. However, oxidative stress also leads to increased concentrations of ROS, which in turn activates a cytoplasmic Ser/Thr kinase, MST1 [39]. Therefore, once the FOXO3-14-3-3 complex is exposed in the cytoplasm, instead of FoxO3 degradation by ubiquitination, MST1 actively phosphorylates FoxO3 at Ser207 [39]. It is quite interesting that this step suggests a competitive interplay between phosphorylation and ubiquitination of human FoxO3 in the cytoplasm, which demands further investigation. Once phosphorylated by MST1, disruption of the FoxO3-14-3-3 complex and translocation of phosphorylated FoxO3 back into the nucleus via importins takes place [39]. Recently, it has been shown that the Ser/Thr phosphatase PP2A dephosphorylates FoxO3 in the nucleus at several sites, including both AKT and MST1 target sites [62]. At this point FoxO3 is back in the nucleus, so it may retain its nuclear activity but dephosphorylation again gives the opportunity to AKT to re-phosphorylate FoxO3. However, based on the methylation potential of FoxO3 identified here, we suggest that targeting Arg248 and Arg250 via PRMT1, phosphorylation by AKT can be blocked. Similar to mouse FoxO1 methylation, activated PRMT1 in the nucleus as a result of oxidative stress will actively methylate Arg248 and Arg250 residues on human FoxO3, masking the neighboring Ser253 and preventing phosphorylation of FoxO3 by AKT. Therefore, this methylation step has the potential to induce prolonged retention of FoxO3 in the nucleus and activation of pro-apoptotic factors such as Bim, leading to apoptosis of cancerous cells.

The residues Arg264 and Arg266 were also identified as potential methylation sites in human FoxO3 (Table 2). Both residues are conserved and possess the typical PRMT1 recognition motif (RXR) in flanking regions. Whether methylation at these residues has the potential to influence phosphorylation of neighboring Ser/Thr/Tyr residues or the activity of FoxO3 remains to be determined. Interestingly, these two residues are in close proximity to Lys residues, which are often modified via acetylation. This suggests interplay between acetylation and methylation may also exist in human FoxO3. Although the methylation potential of human FoxO3 and mouse FoxO1 appears to be an appealing mechanism for blocking phosphorylation via AKT, further investigation is required. Whether methylation at Arg248 and Arg250 will have any influence on phosphorylation via IKK and ERK is still not known.

## 5. Conclusions

Identification of PTM sites forms a foundation for understanding molecular regulatory mechanisms and associated outcomes. The wide distribution of PTMs and the dependent relationship between multiple PTMs strongly implies that crosstalk is a common mechanism and enables the organism to finely tune protein functions at the translational level. The computational findings of this study illustrate the complex and highly dynamic role of FoxO3 and PTMs in human cancers. Our findings are also in consistent with previous studies [11], which showed that FoxO3 is an active target for multiple types of PTMs. Increasing our understanding of PTMs and their crosstalk in FoxO proteins may aid in the development of PTM specific inhibitors. Whether the phosphorylation-*O*-β-glycosylation and phosphorylation-methylation crosstalk in FoxO3 acts as positive or negative regulator in the long term still remains to be confirmed by experimental means. We therefore propose the need to perform directed studies for the elucidation of mechanisms of crosstalks in FoxO3. Furthermore, the availability of full length three-dimensional structure of FoxO3 would certainly increase our understanding of how these target sites influence each other and the overall activity of FoxO3 and protein-protein interactions.

## Figures and Tables

**Table 1:** Computationally predicted and experimentally determined phosphorylation, *O*-β-GlcNAc modifications, and Yin Yang sites in human FoxO3.

Residues			Phosphorylation		Scansite		*O*-GlcNAc		Yin Yang
				
Name	Pos	CS	EV	CD	Kinases	SA	EV	CD	CD
**Serine**	7	*	[38]	Y	-	-	-	Y	Y
	12	*	[38]	Y	ERK1; AKT	0.8	-	-	-
	26	*	-	Y	GSK3	4.8	-	Y	Y
	30	*	-	Y	-	-	-	-	-
	43	*	-	Y	Cdk5	1.7	-	-	-
	48	*	-	Y	-	-	-	Y	Y
	55	*	-	Y	-	-	-	-	-
	110	*	-	Y	-	-	-	Y	Y
	144	*	-	Y	-	-	-	Y	-
	151	*	-	Y	PKC; PKA	1.7	-	Y	Y
	152	*	-	Y	-	-	-	-	-
	161	*	-	Y	-	-	-	-	-
	172	*	-	Y	-	-	-	-	-
	173	*	-	Y	Cdk5; Cdc2	3.6	-	-	-
	200	*	-	Y	-	-	-	-	-
	209	*	[39]	Y	PKC	1.9	-	-	-
	243	*	-	Y	-	-	-	-	-
	253	*	[40]	Y	AKT E; PKA	0.6	-	Y	Y
	257	*	-	Y	-	-	-	-	-
	280	*	[41]	Y	CK2; GSK3	2.3	-	Y	Y
	284	*	[41]	Y	ERK	1.9	-	-	-
	294	*	[5]	Y	Cdk5; Cdc2; ERK1 E, p38 MAPK	1.4	-	-	**FN**
	297	*	-	Y	-	-	-	Y	Y
	299	*	[41]	Y	-	-	-	-	-
	300	*	-	Y	CK2	2.0	-	-	-
	311	*	-	-	-	-	-	Y	-
	315	*	[40]	Y	AKT E, Clk2	1.6	-	Y	Y
	318	*	[42]	Y	CK1 E	1.7	-	-	-
	321	*	[42]	Y	CK1 E	1.9	-	Y	-
	325	*	[43]	Y	ERK1	0.5	-	-	-
	330	*	-	Y	-	-	-	-	-
	344	*	[5]	Y	ERK1 E	0.6	-	Y	Y
	349	*	-	-	-	-	-	Y	-
	350	*	-	-	-	-	-	Y	-
	351	*	-	-	GSK3	0.6	-	Y	-
	353	*	-	Y	CK1; PKC; PKC δ	0.7	-	-	-
	355	*	-	Y	ERK1; Cdk5	0.5	-	Y	Y
	357	*	-	Y	-	-	-	-	-
	359	*	-	Y	-	-	-	Y	Y
	399	*	[44]	Y	ATMK; AMPK E	2.6	-	-	-
	402	*	-	Y	ERK1	1.9	-	-	
	411	*	-	-	-	-	-	Y	-
	413	*	[44]	Y	AKT; PKC	1.1	-	-	-
	421	*	[45]	Y	CK1	0.5	-	Y	-
	425	*	[5]	Y	Cdc2; Cdk5; GSK3; ERK1 E	0.7	-	-	**FN**
	428	*	-	-	-	-	-	Y	-
	429	*	-	Y	-	-	-	-	-
	432	*	-	Y	CK1	0.5	-	-	-
	442	*	-	Y	-	-	-	-	-
	446	*	-	Y	-	-	-	-	-
	463	*	-	Y	CK1	0.9	-	-	-
	476	*	-	-	-	-	-	Y	-
	480	*	-	Y	-	-	-	-	-
	482	*	-	Y	-	-	-	-	-
	494	*	-	Y	-	-	-	Y	Y
	497	*	-	-	PKC	0.6	-	Y	-
	501	*	-	Y	-	-	-	Y	Y
	547	*	-	-	-	-	-	Y	-
	551	*	-	Y	-	-	-	-	-
	553	*	-	Y	PKC	1.2	-	-	-
	560	*	-	Y	-	-	-	Y	Y
	563	*	-	-	-	-	-	Y	-
	564	*	-	Y	-	-	-	-	-
	567	*	-	Y	PKC	0.7	-	-	-
	574	*	-	Y	-	-	-	-	-
	577	*	-	Y	ATMK	0.7	-	Y	Y
	584	*	-	-	-	-	-	Y	-
	586	*	-	Y	-	-	-	-	-
	588	*	[44]	Y	AMPK E	0.6	-	-	-
	591	*	-	Y	PKC; CK1	0.7	-	-	-
	594	*	-	Y	-	-	-	-	-
	609	*	-	Y	-	-	-	-	-
	626	*	[44]	Y	-	-	-	-	-
	644	*	[4]	Y	-	-	-	-	**FN**
	666	*	-	-	-	-	-	Y	-
	667	*	-	Y	-	-	-	Y	Y
	669	*	-	-	-	-	-	Y	-
**Threonine**	32	*	[46]	Y	AKT E, PKA	0.5	-	-	**FN**
	228	*	-	Y	-	-	-	-	-
	276	*	-	-	-	-	-	Y	-
	296	*	-	Y	PKC	2.5	-	Y	Y
	331	*	-	Y	CK2	1.0	-	-	-
	395	-	-	-	-	-	-	Y	-
	404	-	-	-	-	-	-	Y	-
	417	*	-	Y	PKC	2.3	-	-	-
	418	-	-	-	-	-	-	Y	-
	450	*	-	Y	-	-	-	-	-
	469	*	-	Y	-	-	-	-	-
	487	*	-	Y	DNA PK	1.2	-	-	-
	498	*	-	-	-	-	-	Y	-
	540	*	-	Y	-	-	-	-	-
	582	*	-	Y	PKC; CK1	1.0	-	Y	Y
	660	*	-	-	-	-	-	Y	-
**Tyrosine**	162	*	-	Y	Lck kinase	0.6	-	-	-
	260	*	-	Y	-	-	-	-	-
	416	*	-	Y	-	-	-	-	-
	465	*	-	Y	Grb2 SH2	-	-	-	-

Pos: Position; CS: Conservation status;

* Conserved residues;

EV: Experimentally verified; CD: Computationally determined; FN: False negative Yin Yang sites;

E Experimentally confirmed.

**Table 2:** Methylation sites in human FoxO3.

Residues		Conservation Status	Flanking Sequence	Methylation Status	

Name	Position			CD	EV
Arg	248	*	GKSGKAP*R*RR	Y	Based on sequence similarity with mouse FOXO1 methylation sites [54]
Arg	250	*	PRR*R*AVSMD	Y	Based on sequence similarity with mouse FOXO1 methylation sites [54]
Arg	264	*	NKYTKS*R*GRAAKK	Y	-
Arg	266	*	YTKSRG*R*AAKKKA	Y	-
Lys	149	*	GGSGQPR*K*CSSRR	Y	-
Lys	207	*	SNSSAGW*K*NSIRH	Y	-
Lys	270	*	SRGRAAK*K*KAALQ	Y	-
Lys	271	*	SRGRAAKK*K*AALQ	Y	-
Lys	569	*	SSSLGSA*K*HQQQS	Y	-

* Conserved residues;

CD: Computationally determined; Y: Yes; EV: Experimentally verified.
